# Responses of molluscan communities to centuries of human impact in the northern Adriatic Sea

**DOI:** 10.1371/journal.pone.0180820

**Published:** 2017-07-19

**Authors:** Ivo Gallmetzer, Alexandra Haselmair, Adam Tomašových, Michael Stachowitsch, Martin Zuschin

**Affiliations:** 1 Department of Palaeontology, University of Vienna, Vienna, Austria; 2 Geological Institute, Slovak Academy of Sciences, Bratislava, Slovakia; 3 Department of Limnology and Bio-Oceanography, Center of Ecology, University of Vienna, Vienna, Austria; University of California, UNITED STATES

## Abstract

In sediment cores spanning ~500 years of history in the Gulf of Trieste, down-core changes in molluscan community structure are characterized by marked shifts in species and functional composition. Between the 16^th^ and 19^th^ century, a strong heavy metal contamination of the sediments, most notably by Hg, together with the effects of natural climatic oscillations (increased sedimentation and organic enrichment) drive community changes. Since the early 20^th^ century up to 2013, the combined impacts of cultural eutrophication, frequent hypoxic events and intensifying bottom trawling replace heavy metal contamination and climatic factors as the main drivers. The pollution-tolerant and opportunistic bivalve *Corbula gibba* and the scavenging gastropod *Nassarius pygmaeus* significantly increase in abundance during the 20^th^ century, while species more sensitive to disturbances and hypoxia such as *Turritella communis* and *Kurtiella bidentata* become rare or absent. An infaunal life habit and scavenging emerge as the dominant life strategies during the late 20^th^ century. Down-core shifts in the proportional abundances of molluscan species and functional groups represent a sensitive proxy for past ecological changes and reveal a century-long anthropogenic impact as the main driver behind these processes in the northern Adriatic Sea, offering also a unique perspective for other shallow marine ecosystems worldwide.

## Introduction

Most marine ecosystems worldwide are affected by human activities, and pristine coastal areas have virtually disappeared [1–3]. The human influence ranges from direct exploitation of multiple resources to pollution, from coastal urbanisation to eutrophication, and involves large-scale and complex phenomena such as marine invasions, ocean acidification and global warming. Modern ecological studies often fail to capture long-term ecosystem responses to anthropogenic stressors because they are restricted to a time scale of a few years or a few decades and thus do not recover conditions pre-dating anthropogenic disturbance. An approach integrating palaeontological, sedimentological and geochronological methods applied to sediment cores [4, 5] and including the study of historical accounts relevant to the topic can solve this problem and discriminate between natural and anthropogenic drivers of changes in benthic ecosystems [6].

The northern Adriatic Sea is ideal for this type of investigation due to its long history of human interactions with the marine environment and comparatively long written historical documentation. The coasts of this shallow epicontinental sea have been densely urbanised since historical times, and the many rivers discharging into the basin—above all the Po River—enrich it with nutrients and pollutants [1, 7]. The Gulf of Trieste with the Bay of Panzano in its northernmost part is particularly affected due to its very shallow waters, several industrial port areas, mariculture infrastructures, and intense ship traffic [8, 9]. Additionally, the Bay of Panzano has a unique history of heavy metal contamination driven by the mercury mine of Idrija in the Slovenian hinterland, which was active from the late 15^th^ century until a few decades ago [10, 11].

Several studies from the north-western Adriatic (mainly from the Po River Delta) documented the stratigraphic changes in foraminiferal and mollusc communities [12–14] and quantified community shifts between Late Pleistocene, Holocene and modern assemblages [15]. Kowalewski et al. [15] identified significant compositional changes between Holocene and 20^th^ century molluscan death assemblages, most likely due to anthropogenic impact, whereas Pleistocene and Holocene interglacial communities were surprisingly similar. Most studies estimating the effects of anthropogenic disturbances in the Gulf of Trieste focused either on geochemical [8, 16, 17] or biological markers [18]; some of them followed a more integrated approach [19–21]. All, however, are limited in their historical perspective to the present or a relatively recent past and thus do not assess community states preceding the 20^th^ century conditions in the Gulf. Recently, changes in the composition of foraminiferal communities [22] and in abundance of the opportunistic bivalve *Corbula gibba* [23] over the past few centuries in the Gulf of Trieste were evaluated based on the same sediment cores used for the present study. Vidović et al. [22] concluded that long-term organic enrichment in this area shaped foraminiferal communities, with species adapted to high nutrient supply and tolerant to hypoxia increasing in abundance in the late 20^th^ century. Tomašových et al. [23] found that production of the opportunistic bivalve *Corbula gibba* strongly fluctuated over the pasts few centuries, and suggested that intervals with higher frequency of hypoxia were not exclusively driven by human-induced enrichment in the 20^th^ century. However, so far, baseline states and long-term responses of whole macrobenthic communities to natural or anthropogenic impacts in the Gulf of Trieste remain largely unknown.

Here, we focus on down-core changes in species and guild composition of mollusc communities (focusing on two key functional aspects—feeding and substrate relation) to trace past environmental shifts over the past few centuries. Molluscs can respond differently to external stressors compared to protists, thus capturing aspects of the environmental history not well represented in the foraminiferal record. Moreover, this macrofauna group has the highest preservation potential, with species that range from very sensitive to very resilient [24]. Hence, molluscs are frequently used as representatives for whole macrofaunal assemblages [6, 25, 26]. Our investigation is based on the molluscan shell record of two 1.5-m-long sediment cores from Panzano Bay, covering about 500 years, and is supplemented by geochemical and geochronological analyses. Molluscan death assemblages in these cores are time-averaged due to bioturbation and other sediment mixing processes [23], meaning that individual stratigraphic increments contain a mixture of shells of different age encompassing a time span of decades or one-two centuries. Post-mortem shell transport could bias the composition of death assemblages, but it has been shown that even in high-energy environments it does not tend to homogenize macrobenthic communities on a larger spatial scale [25, 27]. In Panzano Bay, however, out-of-habitat transport as well as shell dissolution, another biasing factor, are negligible [28, 29]. Time-averaging has the effect that in death assemblages, short-term community fluctuations occurring at seasonal or yearly scales (e.g. due to changing across-year recruitment patterns) are averaged out. The significant compositional changes in our cores, therefore, are likely related to major environmental shifts occurring over longer time spans.

The direct dating of mollusc shells and the geochemical analysis enabled us to relate community changes to specific time periods, abiotic factors and human interferences as determined by the historical literature. We evaluate a) whether concentrations of nutrients, heavy metals and organic pollutants in the sediment increase up-core; and b) whether heavy metal contamination, eutrophication, bottom trawling, and hypoxic events affect molluscan community composition and lead to an up-core decline in diversity and abundance of disturbance-sensitive species. We provide a well-resolved historical record of environmental changes in the northern Adriatic Sea and highlight the relative importance of specific forms of anthropogenic disturbance in altering the mollusc community.

## Material and methods

### Study area

The shallow northern Adriatic Sea originated at the onset of the Holocene when the rising sea level gradually covered the vast alluvial plains that had been exposed since the Würmian glaciation (18,000–23,000 BC) [30]. The Bay of Panzano is a large embayment of the Gulf of Trieste to the north-west and was formed by the progressive deposition of mostly carbonate material by the Isonzo (Soča) River [31]. The sediments of the bay are formed by sublittoral muds that fill much of the basin. The river delta features pebble-enriched sands in the shallow nearshore areas and an increasing pelitic fraction in the delta foreset beds [17, 29]. The hydrography of Panzano Bay is characterized by the interaction of tides (semi-diurnal, average tidal range ~ 0.5 m), the confluence of the Isonzo River (average discharge between 100–130 m^3^/s [31]), and strong winds. Winds and freshwater influx from the Isonzo River are subject to considerable seasonal variations. The river discharge is characterized by two flood periods, one in spring due to snowmelt, and the other in autumn, caused by heavy rainfalls [32]. North-easterly bora winds prevail in winter, and south-westerly sirocco winds dominate during summer months [33]. Due to the bay’s shallow depth (<15 m), strong winds are likely to re-suspend surface sediments, thus leading to a marked and persistent water turbidity [33, 34]. This is further augmented by the intensive traffic of large cargo ships and oil tankers whose propellers affect the fine sediments on the bottom [35]. In many respects (water depth, sediment type, recent mollusc community composition, stratigraphic units), the location of the sampling station in Panzano Bay at 12 m depth reflects conditions characterizing a large part of the Gulf of Trieste [18, 36–39]. Over the time period covered by the cores (about 500 years), relative sea level changes stayed within the range of ~– 0.5 m [40], suggesting that water-level fluctuations (otherwise an important community-structuring factor to consider in sedimentary successions [13]) are negligible in their influence on environmental conditions in the area.

### Anthropogenic impacts

One of the main sources of pollution is the large industrial port of Monfalcone with a thermoelectric plant and numerous coal-, petroleum- und cargo-handling piers, founded in the 19^th^ century and today one of the most important port areas on the Italian Adriatic coast. Zones with intense mussel and fish farming occur along the NE coast of the bay. The Isonzo River is a major source of organics and mercury pollution. The mercury mine of Idrija, located about 100 km from the sea in the Slovenian hinterlands, was established in the late 15^th^ century and was shut down only in 1995. During the centuries-long intense mining activities, more than five million tons of Hg ore were excavated [41]. Most of the mining and smelting residues were dumped along the banks of the Idrijca River and eventually washed into the Gulf of Trieste [11, 17]. In surface sediments, Hg contamination is still extensive at present, especially near the Isonzo delta, where concentrations reach up to 14 mg/kg [17]. The Gulf of Trieste is also one of the areas in the Adriatic with the highest incidence of mucilage and hypoxic events [42, 43]. Favoured by eutrophic conditions and stable water column stratification in the warm season, the frequency of these events increased during the 20^th^ century in the whole Northern Adriatic Sea, leading to widespread die-offs of benthic organisms [44–47]. These human impacts are compounded by the effects of a long history of fishing, which was already intense during past centuries [48, 49] but experienced a boost in the 20^th^ century with the industrialisation of the sector [1, 50]. Especially modern bottom trawling led to a strong decline in many fish species and altered benthic communities [51–54].

### Sampling and analyses

**Fig 1:**
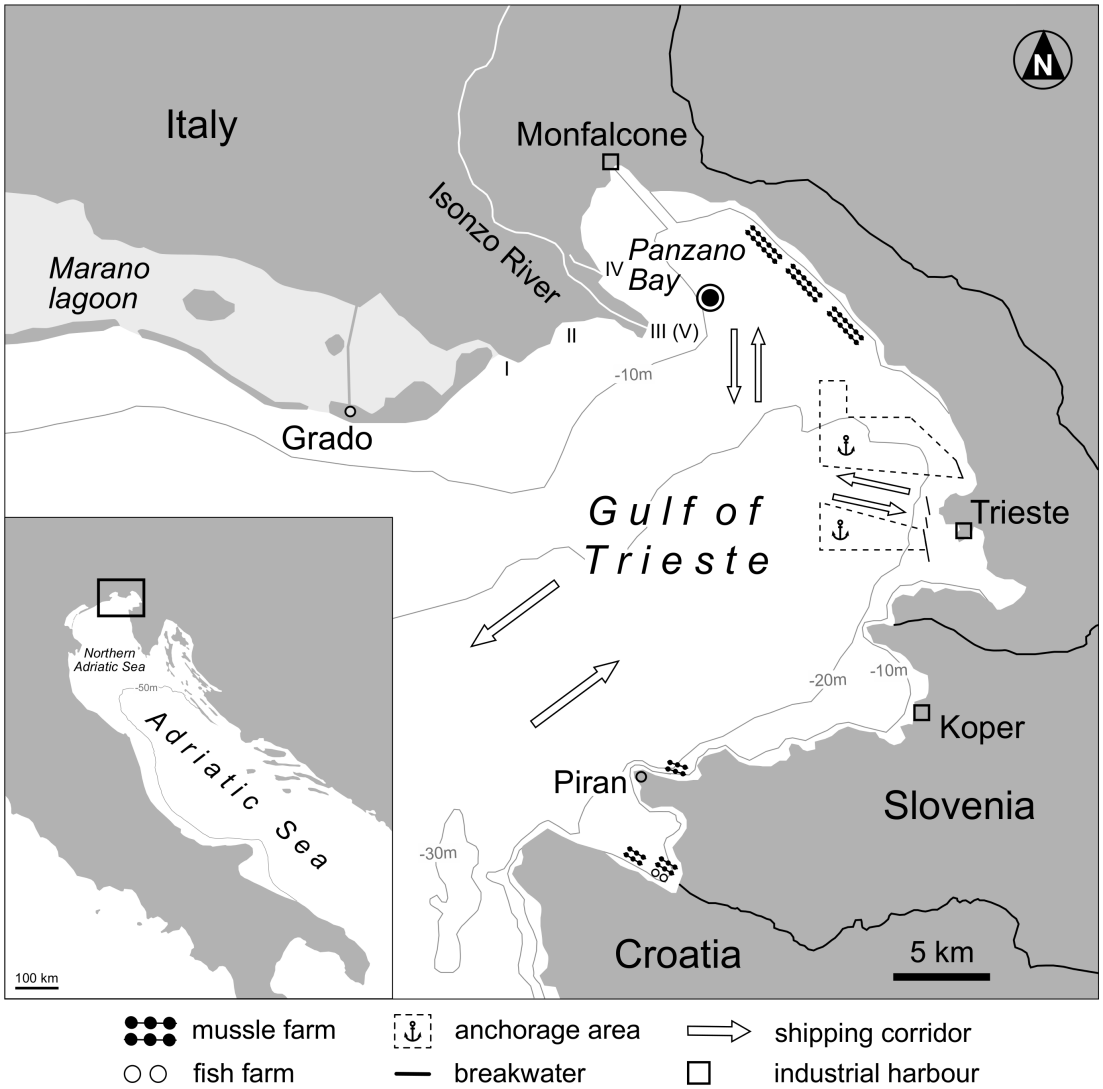
Location of sampling station in Panzano Bay, north-western Gulf of Trieste, Adriatic Sea. Roman numerals I–V along the coast between Grado and Monfalcone indicate Isonzo River mouth migration stages from Roman times up to the present, according to Brambati [56]: I: 313- ~600 AD, II: ~600–1589 AD, III: 1589–1896 AD, IV: 1896–1937 AD, V: after 1937 AD. The Gulf of Trieste features a layered gyre-type circulation pattern with a permanent cyclonic current in the bottom layer and an alternating cyclonic/anticyclonic flow on the surface [57].

At a 12-m-deep station in the middle of the bay (coordinates: N 45° 44' 7.44" / E 13° 36' 1.68"), in August 2013, four sediment cores were taken within a few meters of each other using an UWITEC^®^ piston corer with hammer action [55] (Fig 1). The two cores with 16-cm diameter (M28 and M29) were used for the analyses of molluscan death assemblages, the two 9-cm-diameter cores (S25 and S27) for ^210^Pb sediment dating, granulometry, and the analysis of heavy metal, nutrient and organic pollutant concentrations. Foraminiferal communities in core M29 were analysed by Vidović et al. [22], while for age dating *C*. *gibba* shells from M28 and M29 were subjected to radiocarbon-calibrated amino acid racemization by Tomašových et al. [23].

#### Molluscan community analysis

The molluscan cores were sliced on board into smaller subsamples: 2-cm increments for the uppermost 20 cm, and 5-cm-increments for the rest of the core. This subdivision was originally chosen to obtain a higher resolution for surficial core strata potentially more intensively affected by anthropogenic impacts. However, the thickness of the surface mixed layer (6 cm) and the decadal scale of time-averaging shows that pairs of adjacent 2-cm increments can be pooled to obtain a better comparability with the thicker 5-cm increments used for the segmentation of the bulk of the core. In the lab, these subsamples were sieved through a 1-mm mesh size and all the molluscan shells were picked from the dried material under a stereomicroscope. The shells were then counted and determined to species level. To consider a shell fragment as a valid specimen, in bivalves the hinge and in gastropods the apex had to be present. For bivalve species, abundance was calculated by adding the number of double-valved specimens to whichever number of single valves (right or left) was higher. In chitons, abundance was estimated by adding the higher number of terminal plates (either cephalic or anal plates) to the number of dorsal plates divided by six. For scaphopods, the number of entire individuals was added to the higher number of apex or base fragments, while intermediate shell pieces were only counted as individuals if they differed clearly in size from all the other fragments in a sample. We assigned every mollusc species in our dataset to functional categories with regard to feeding guild, organism-substrate relation, and host association (Table 1; categories after Rueda et al. [58]; see also S1 Dataset). We define “host association” as any kind of parasitic, commensal or mutualistic relationship between mollusc species and other animal taxa, including molluscs.

**Table 1:** Functional groups.

Feeding guild	Organism—substrate relation	Host association
herbivore	infauna	no association
• *herbivore*	• *soft bottom infauna*	ascidians
• *herbivore/detritivore*	• *soft bottom semi-infauna*	molluscs
detritivore	• *hard bottom infauna (borers)*	echinoderms
• *detritivore*	epifauna	crustaceans
• *detritivore/filter feeding*	• *soft bottom epifauna*	sponges
filter feeding	• *hard bottom epifauna*	unknown
carnivore	• *epifauna soft & hard bottoms*	
grazing	• *epifauna on vegetation*	
scavenging	soft bottom nestler	
chemosymbiotic/filter feeding	epibionts and ectoparasits	
	terrestrial	

Overview of functional categories characterizing the life habit of the found mollusc species. Table columns are lists of functional categories and are independent of each other; there is no connection between categories within rows. Sub-categories with bullet-points and in italics.

#### Granulometry

The core S25 designated for granulometry and sediment dating was sliced on board following the same procedure as for the molluscan core. In the lab, from each core increment, a small subsample (10–20 g) was taken to analyse grain size distribution. These samples were screened through a set of five sieves (1 mm, 0.5 mm, 0.250 mm, 0.125 mm and 0.063 mm). The individual fractions were dried and weighed, and the fraction < 0.063 mm was then used for granulometric analysis with a SediGraph III 5120 Particle Size Analyzer. Four grain size classes were defined: clay (< 0.002 mm), silt (0.002–0.063 mm), sand (0.063–2 mm) and gravel (> 2 mm), the latter two categories consisting mostly of shells, shell fragments and other biogenic material.

#### Geochemistry

The other 9-cm-diameter core was sealed immediately after retrieval and further processed at the ISMAR institute, Venice. In a first step, a radiographic image of the whole core was taken to check for down-core sediment density trends that help identify the most convenient intervals for sample extraction and analysis. The following intervals were selected: 0–2 cm, 4–6 cm, 8–10 cm, 23–25 cm, 45–47 cm, 68–70 cm, 84–86 cm, 104–106 cm, 125–127 cm and 150–152 cm. For each of these intervals, three groups of parameters were measured: concentration of metals (Hg, Cu, Cr, Ni, Pb, As, Cd, Zn in mg/kg), concentration of nutrients (total carbon, total organic carbon, and total nitrogen in percentage of dry weight) and concentration of organic pollutants (polychlorinated biphenyls “PCBs” and polycyclic aromatic hydrocarbons “PAHs” in ng/g). For a detailed analytic protocol, refer to Vidović et al. [22]. The concentrations found were checked against threshold levels for polluted sediments provided by NOAA and the Italian government.

#### Sediment and shell dating

**Fig 2:**
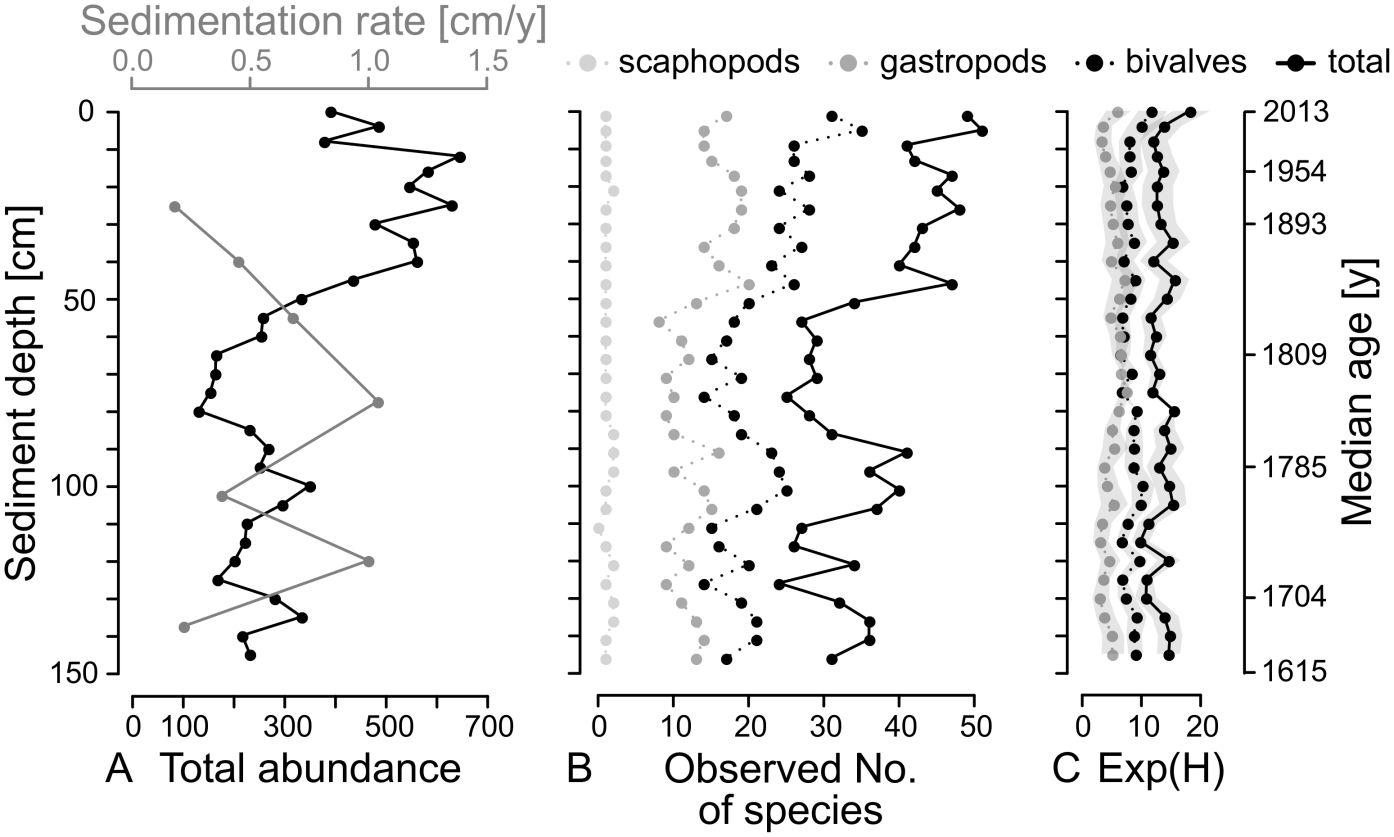
Total mollusc abundance, species richness, and diversity. Down-core trends in abundance, species richness, and diversity of bivalves, gastropods and scaphopods in the Panzano Bay sediment core. Species richness is given as observed number of species, and diversity as the exponent of the Shannon index, exp(H) (effective number of species). Overall down-core abundance (left graph) is plotted together with sedimentation rates as derived from median *C*. *gibba* shell ages of dated sediment layers.

Core chronology is based on (1) ^210^Pb sediment dating of the upper 40 cm and (2) radiocarbon-calibrated amino acid racemization (AAR) dating of the bivalve species *C*. *gibba*. The AAR rate in 311 shells of this species was calibrated by ^14^C ages of eleven dead and three live-collected specimens, using the approach of Allen et al. [59]. The details on ^210^Pb sediment dating, on AAR and ^14^C measurements, and on the AAR calibration method applied for the shells are described in Tomašových et al. [23]. Concentrations of ^210^Pb-excess are uniform in the upper 6 cm (surface mixed layer), and monotonically decline to background values at 30 cm. Sedimentation rates estimated for the upper 30 cm from the slope of the decay in excess ^210^Pb correspond to 0.24 cm/year (95% conf. intervals = 0.2–0.28 cm/year). Increment ages based on radiocarbon-calibrated AAR in *C*. *gibba* shells are congruent with ^210^Pb ages, and show that increments are time-averaged (with time-averaging measured as an inter-quartile range of *C*. *gibba* ages in each increment) to 10–20 years in the upper 20 cm up to ~100–200 years in the middle and lower parts of the core [23]. Here, we group increments with similar median ages and strongly overlapping age distributions into six temporal bins: 150–130 cm (16^th^-17^th^ century), 130–95 cm (18^th^ century), 95–65 cm (early 19th century), 65–30 cm (late 19th century), 30–16 cm (1900–1950 AD) and 16–0 cm (1950–2013). AAR data imply that the net sedimentation rate was about 0.2 cm/year during the 20^th^ century and 0.48 cm/ year before the 20^th^ century [23]. A finer resolution of pre-20^th^-century sedimentation rates was obtained by dividing the temporal separation between median shell ages of dated layers by the corresponding sediment thickness (Fig 2A).

#### Statistical analysis

Environmental variables include percentages of four grain-size categories (clay, silt, sand, gravel), concentrations of total organic carbon (TOC) and total nitrogen (Ntot), organic pollutants (PCBs, PAHs), and eight elements (Hg, Zn, Cr, Cu, Ni, Pb, As, Cd). Non-normally distributed environmental variables were log-transformed, and all variables were z-standardized due to different units and scales. Covariance-based Principal Component Analysis (PCA) was used to identify relations between concentrations of environmental variables and sediment depth.

In analyses of molluscan community composition and diversity, 2-cm-thick increments in the top 20 cm were pooled into 4-cm-thick increments, and increments of equivalent sediment depths in two cores (M28 and M29) were pooled into a single composite core. Species diversity is expressed a) as the number of recorded species; b) as the exponential of Shannon entropy (exp(H)), the “true” diversity or “effective number of species”. This diversity measure transforms standard diversity indices onto a linear, easy-to-visualize scale representing the number of species in perfectly even communities [60]. Differences in effective species richness between six temporal bins were tested using the Wilcoxon rank test and the median number of species. Differences in molluscan community composition between consecutive bins were quantified by the pseudo-F statistics of permutational multivariate analysis of variance (PERMANOVA), and its significance assessed by randomization of increment attribution to particular temporal bins. This statistics represents the ratio between the variation explained by temporal bins and the unexplained variation. Non-metric multi-dimensional scaling (NMDS) based on Bray-Curtis distances and fit to two dimensions was used to visualize changes in the mollusc community structure down the sediment core (no rare species or singletons were removed). This ordination method is a powerful tool to examine gradients in species composition [15, 61]. Bray-Curtis distances are based on square-root transformed relative abundances. Stress values <0.2 provide a useful two-dimensional representation of variation in community composition [62]. Correlations between NMDS-axes scores and environmental factors enabled us to explore the sources of compositional variation in ordination space. Redundancy analysis (RDA) based on a forward selection model was applied to quantify the amount of community variation explained by environmental variables (“ordi2step”–function). This procedure was performed only for those core intervals for which measurement data of environmental factors are available. Using square-root transformed relative abundances (i.e. Hellinger transformation), compositional distances among mollusc assemblages in RDA ordinations tend to accurately preserve Euclidean distances among them [63]. All statistical analyses were performed in R-studio (version 3.1.3.) using the “vegan” package.

## Results

### Sedimentology

**Fig 3:**
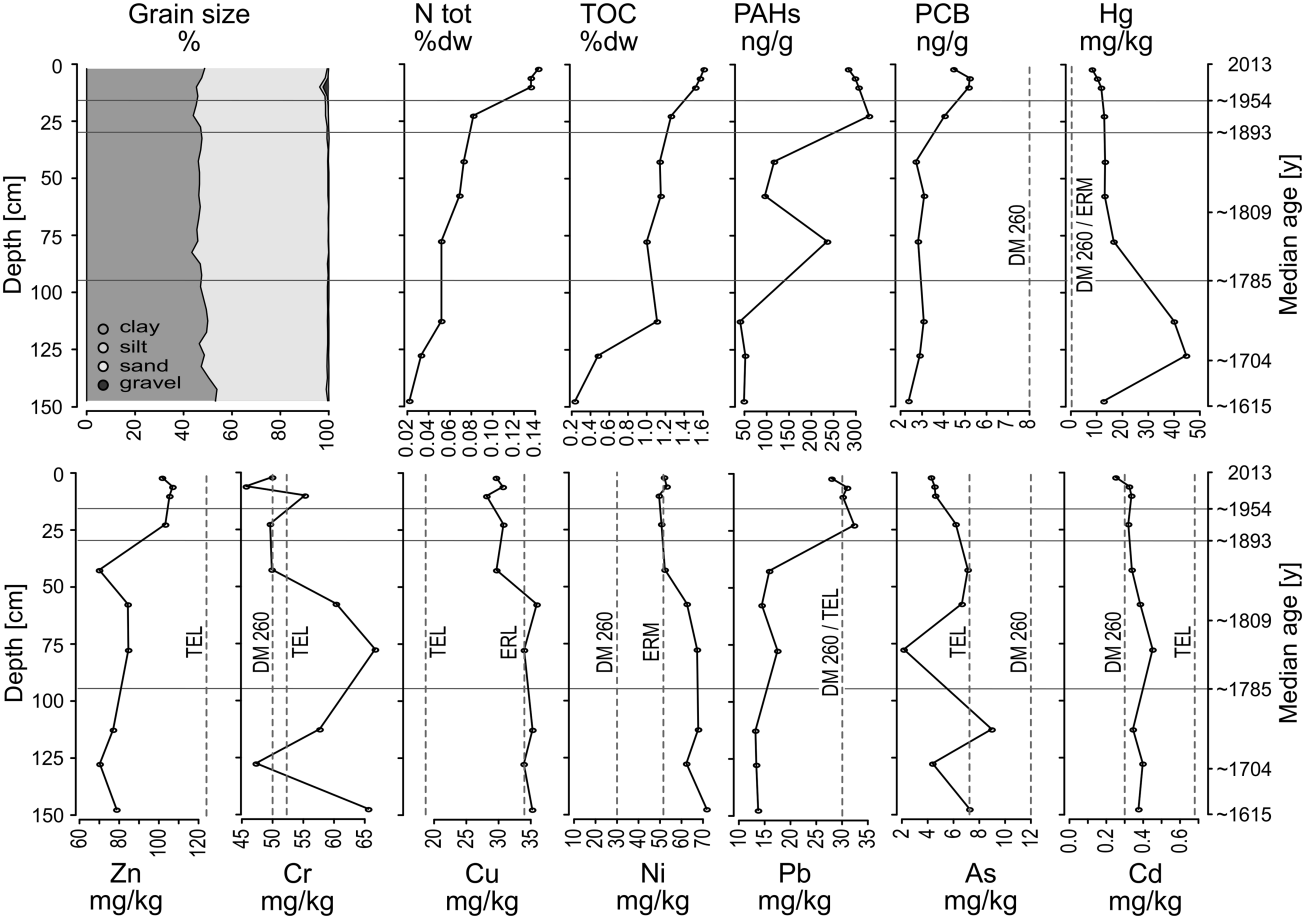
Grain size analysis and concentrations of several metals, nutrients and organic pollutants. Dashed lines indicate threshold values for potential environmental effects according to NOAA sediment quality guidelines and to the Italian Ministerial Decree 26/2010 (ERL: Effects Range-Low; ERM: Effects Range-Median; TEL: Threshold Effects Level). For PAHs, no thresholds are plotted because measured concentrations are considerably lower.

The sediment composition in Panzano Bay is very homogeneous throughout the cores. The sediment consists mainly of clay and silt in almost equal parts, with sand and gravel contributing less than 2%. In the uppermost 20 cm, the coarser fractions increase up to 4% (10–12 cm), mainly due to a higher abundance of serpulid tube fragments (Fig 3 and S2 Dataset).

### Geochemistry

**Fig 4:**
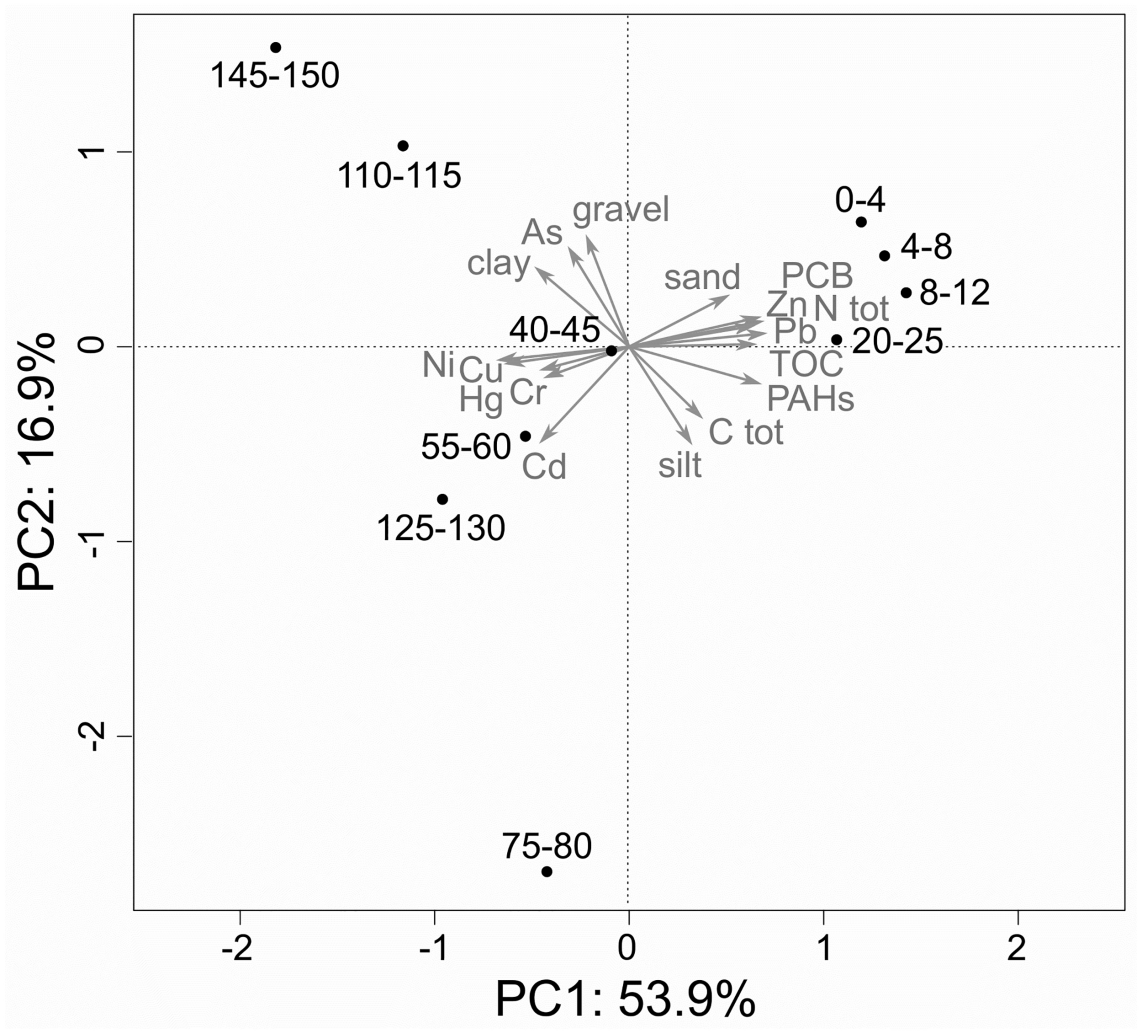
PCA of the investigated metal, nutrient and organic pollutant concentrations.

Down-core concentrations are highly variable for the investigated heavy metals, nutrients, and organic pollutants (Fig 3 and S2 Dataset). Two groups of geochemical variables showing distinct stratigraphic distribution are evident in the PCA (Fig 4). On one hand, concentrations of Pb, Zn, PAH, PCB, TOC, Ntot, and sand percentage are low in the deepest core layers, increase slightly in the intermediate section and rise to peak values in the uppermost sediment layers, starting from about 35 cm core depth (Fig 3). On the other hand, Cu, Cr, Ni and Hg decrease towards the surface and reach minimum values in the upper 35 cm. Concentrations of Cd follow a similar trend, with values decreasing in the upper 75 cm, but remaining high throughout the lower part. The concentrations of all heavy metals (except Cr) and organic pollutants drop conspicuously in the uppermost 5 cm of sediment. Hg concentration peaks at 127.5 cm core depth, then decreases until 75 cm depth, and remains at a constant level almost up to the surface, where it further drops. Some heavy metal concentrations markedly exceed pollution thresholds (Fig 3). The peak concentration of Hg (44.7 mg/kg) is more than 50 times the PEL or ERM values of the NOAA sediment quality guidelines and the lowest concentrations in the surface sediments (8.22 mg/kg) are 10 times higher than threshold values. Only the concentrations of PAHs and PCBs remain below critical values in the whole core. Organic pollutants, nutrient concentrations (TOC and Ntot) and the heavy metals Cu, Ni, Pb, and Zn contribute to the first PC (56% of variance), with factor loadings > 0.70. The second PC, explaining 15% of total variance, is characterized by high loadings of the silt and clay variables, thus summarizing a grain-size gradient. Hg loads mainly on the third PC, which explains 10% of total variance (Fig 4).

### Molluscan community analysis

#### Absolute and relative abundances

**Fig 5:**
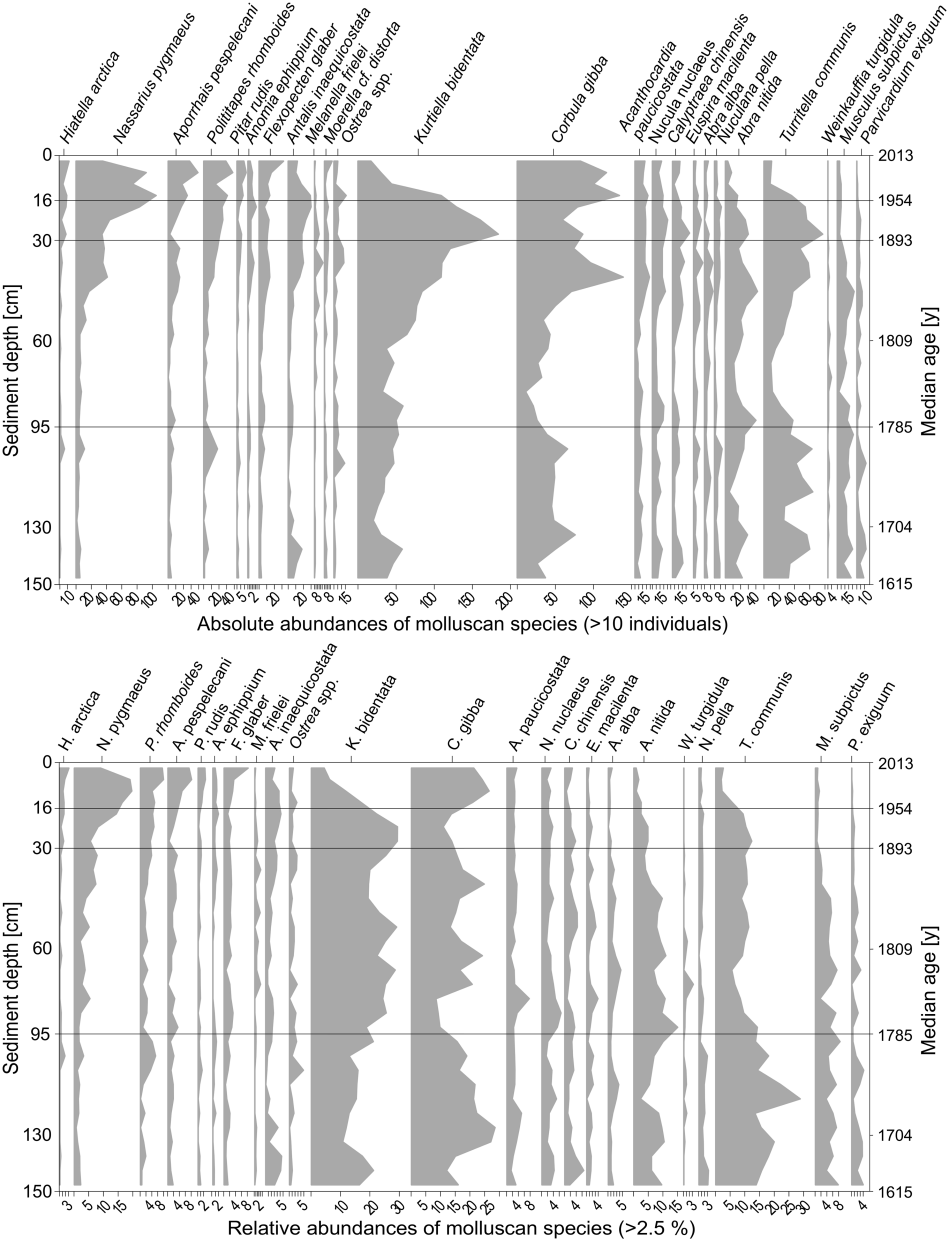
Absolute (>10 individuals) and relative (>2.5%) abundances of the most common mollusc species. Species are sorted according to their weighted average occurrence along the cores, from top (left side) to bottom (right side). Exceptions: maximum occurrence of *Weinkauffia turgidula*: 5; relative abundance of *Pitar rudis*: 2.5%, and of *Anomia ephippium*: 1.9%. For some species, the 0-value of the x-axis has been omitted for better readability.

In total, the two sediment cores contained 7189 bivalve individuals (50 species), 2962 gastropods (50 species), 299 scaphopods (3 species), and one polyplacophoran (see S1 Dataset for a full list of species with down-core abundances). The most abundant bivalves are the commensal galeommatid *Kurtiella bidentata*, the shallow-burrowing suspension-feeders *C*. *gibba* and *Polititapes rhomboides*, and the deep-burrowing deposit feeder *Abra nitida*. The gastropod community is dominated by *Turritella communis*, *Nassarius pygmaeus*, and *Aporrhais pespelecani*. *Antalis inaequicostata* is the most abundant scaphopod. Total molluscan abundance in 4-5-cm thick increments is low in the deeper part of the cores (90–150 cm), oscillating around a mean of 253 individuals per increment, with the smallest abundances in the middle part (early 19^th^ century) and a minimum value of 132 shells in the 80-85-cm increment. Higher up in the cores, abundance increases steeply and peaks at 646 shells between 12 and 16 cm. A sudden drop to almost half this value characterizes the transition to the shallowest sediment layers (Fig 2). This overall abundance fluctuation negatively correlates with sedimentation rates (Fig 2). Several species increase in relative abundance up-core (*N*. *pygmaeus*, *A*. *pespelecani*, *P*. *rhomboides*); most species, however, decrease (*T*. *communis*, *A*. *nitida*, *Musculus subpictus*, *Parvicardium exiguum*), and two species fluctuate but on average remain frequent (*C*. *gibba*, *K*. *bidentata*) (Fig 5).

**Fig 6:**
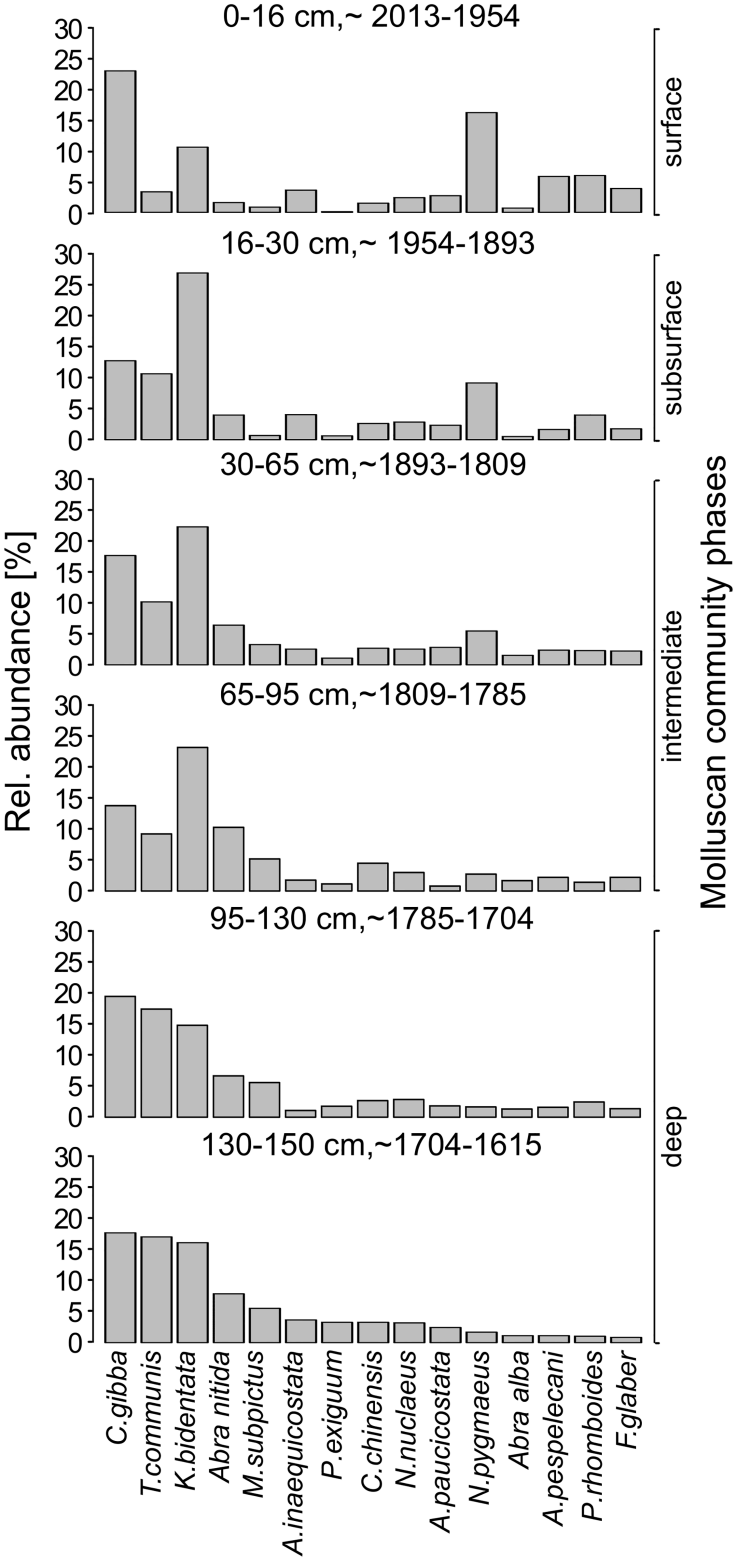
Relative abundance of dominant mollusc species in six temporal bins. Species are sorted according to their abundance rank in the deepest core section.

**Fig 7:**
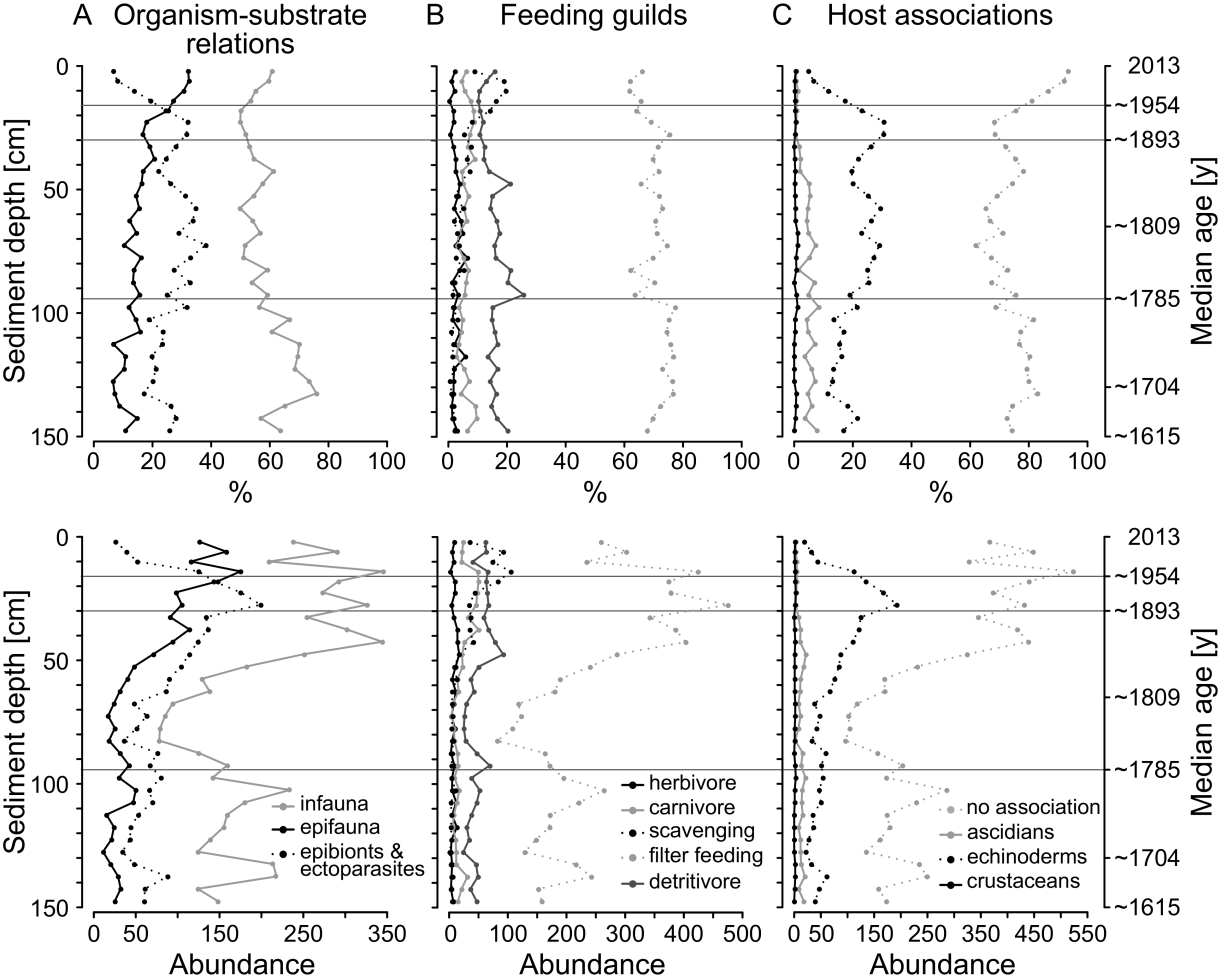
Relative and absolute abundances of molluscs for each functional group. Only main categories are shown (see Table 1), and categories with low abundances are omitted for better readability. Percentages of epifaunal molluscs (left column), scavengers (middle column), and echinoderm commensals (right column) increase while infauna and ascidian commensals decline towards the 20^th^ century. Absolute abundances of infauna also increase towards the 20^th^ century, in contrast to relative abundances.

The first marked temporal changes in community structure based on species relative abundances occur in the 65-90-cm layer (late 18^th^ century) with a strong increase of *K*. *bidentata* and a decrease of *T*. *communis* (Fig 6). This trend continues up the core (max. abundance of *K*. *bidentata* in the early 20^th^ century) and is accompanied by a steady increase of the opportunistic species *N*. *pygmaeus*, which becomes the dominant gastropod in the last 50 years, a period which is characterized by an overall strong compositional and functional shift (Fig 7). The opportunistic bivalve *C*. *gibba*, whose population undergoes moderate fluctuations throughout the core, reaches its maximum relative abundance of 23% in the late 20^th^ century. Formerly abundant species such as *T*. *communis*, *K*. *bidentata*, *A*. *nititda* and *M*. *subpictus* drop considerably or disappear almost completely (*P*. *exiguum*). The molluscan community significantly differs between three pairs of consecutive temporal bins: 0–16 vs 16–30 cm, 16–30 vs 30–65 cm, 65–95 vs 95–130 cm (Table 2). Other pairs of temporal bins do not differ significantly in composition (95–130 vs 130–150 cm; 30–65 vs 65–95 cm) and are pooled in analyses of diversity. Thus, four distinct phases of molluscan community states can be distinguished: the deep section (17-18^th^ centuries, 150–95 cm), the intermediate section (19th century, 95–30 cm), the subsurface section (early 20^th^ century, 30–16 cm), and the surface section (late 20^th^ century. 16–0 cm).

**Table 2:** Differences between consecutive temporal bins with regard to species composition and functional groups, based on PERMANOVA.

temporal bins	species		feeding guild		organism-substrate relation		host association	
	F	Pr(>F)	F	Pr(>F)	F	Pr(>F)	F	Pr(>F)
0–16 vs 16–30 cm	3.286	**0.038**	2.751	0.096	4.743	**0.048**	11.378	0.06
16–30 vs 30–65 cm	2.338	**0.036**	3.983	**0.036**	1.273	0.264	4.362	**0.012**
30–65 vs 65–95 cm	1.594	0.064	1.825	0.131	1.404	0.249	2.053	0.168
65–95 vs 95–130 cm	2.473	**0.001**	2.462	**0.026**	4.237	**0.005**	6.899	**0.005**
95–130 vs 130–150 cm	0.911	0.575	1.918	0.094	0.595	0.688	0.655	0.599
16–30 vs 30–95 cm	3.144	**0.005**	5.658	**0.001**	1.870	0.135	4.485	**0.006**
30–95 vs 95–150 cm	3.799	**0.001**	3.374	**0.004**	8.122	**0.001**	10.497	**0.001**

Listed are F-statistics and p-values from permutation tests. Significant differences in bolt. Last two rows show differences between merged temporal bins 30–95 cm and 95–150 cm.

#### Species richness

Raw molluscan species richness ranges between 23 (125–130 cm core depth, 18^th^ century) and 51 (4–8 cm, late 20^th^ century) species per increment and slightly increases from the bottom of the core to the surface, with a marked low in the middle part (50–90 cm, 19^th^ century, Fig 2B). The observed number of species changes with total abundances. Bivalves generally display higher species numbers than gastropods. The effective species richness (exp(H)) remains relatively constant throughout the cores except for the uppermost layers, where a sharp increase is visible in the 1970s (Fig 2C and 2D). Differences between the effective species richness of the four temporal bins, however, are weak and insignificant except for gastropods where deep and intermediate core section differ significantly (Wilcoxon rank-sum test, p = 0.0001).

#### Functional groups

Filter feeders are the dominant feeding guild (62–77%), followed by detritivores (10–26%), scavengers (0.6–20%), carnivores (3–10%) and herbivores (0.3–6%). The percentages of the individual guilds remain largely constant throughout the core. There are, however, two peaks in detritivores at 95 and 50 cm, respectively, with corresponding lows of filter feeders. From 30 cm sediment depth upwards, a major shift can be observed, with scavengers becoming the second most important feeding guild instead of detritivores, while filter feeders decrease. At 12 cm, this trend reverses again (Fig 7).

The assemblages are dominated by infaunal species throughout the core (50–76%), followed by epibionts and ectoparasites (17–38%), which display a slight increase up until 20 cm sediment depth (early 20^th^ century), and drop to 7% in the late 20^th^ century. This negative trend is compensated by a rise of epifaunal species to a maximum of 32% in the uppermost layer (late 20^th^ century, Fig 7), primarily due to soft-bottom epifaunal species such as *N*. *pygmaeus* and *Flexopecten glaber*. Relative abundances of infaunal species decline upwards, whereas their absolute abundances strongly increase in the upper third of the core (19–20^th^ century).

Abundances of host-associated molluscs, which can represent proxies for occurrences of other taxa such as echinoderms and ascidians, range between 7 and 38%. The largest group with twelve species is associated with echinoderms and predominantly includes *K*. *bidentata* and the eulimid *Melanella frilei* as the most abundant representatives. Their abundances slightly increase up-core until 20 cm depth where they drop, and decline to a minimum of 5% in the surface sediments. A similar trend characterizes ascidian-associated molluscs (mainly *M*. *subpictus*) although their decline already starts at 40 cm core depth. Crustacean- and mollusc-associated species are rare (Fig 7).

Differences between the temporal bins emerge not only with regard to species composition, but also on a functional level (Table 2). For feeding guild and host association, significant differences appear between deep and intermediate (30–95 vs 95–150 cm) and intermediate and subsurface (16–30 vs 30–95 cm) core sections following the pattern found for compositional differences. Organism-substrate relations behave slightly differently; here, changes are most marked in the transition from deep to intermediate and from subsurface to surface (16–30 vs 0–16 cm) section. Feeding guilds and host associations do not change significantly within the uppermost 30 cm.

#### Community shifts through time

**Fig 8:**
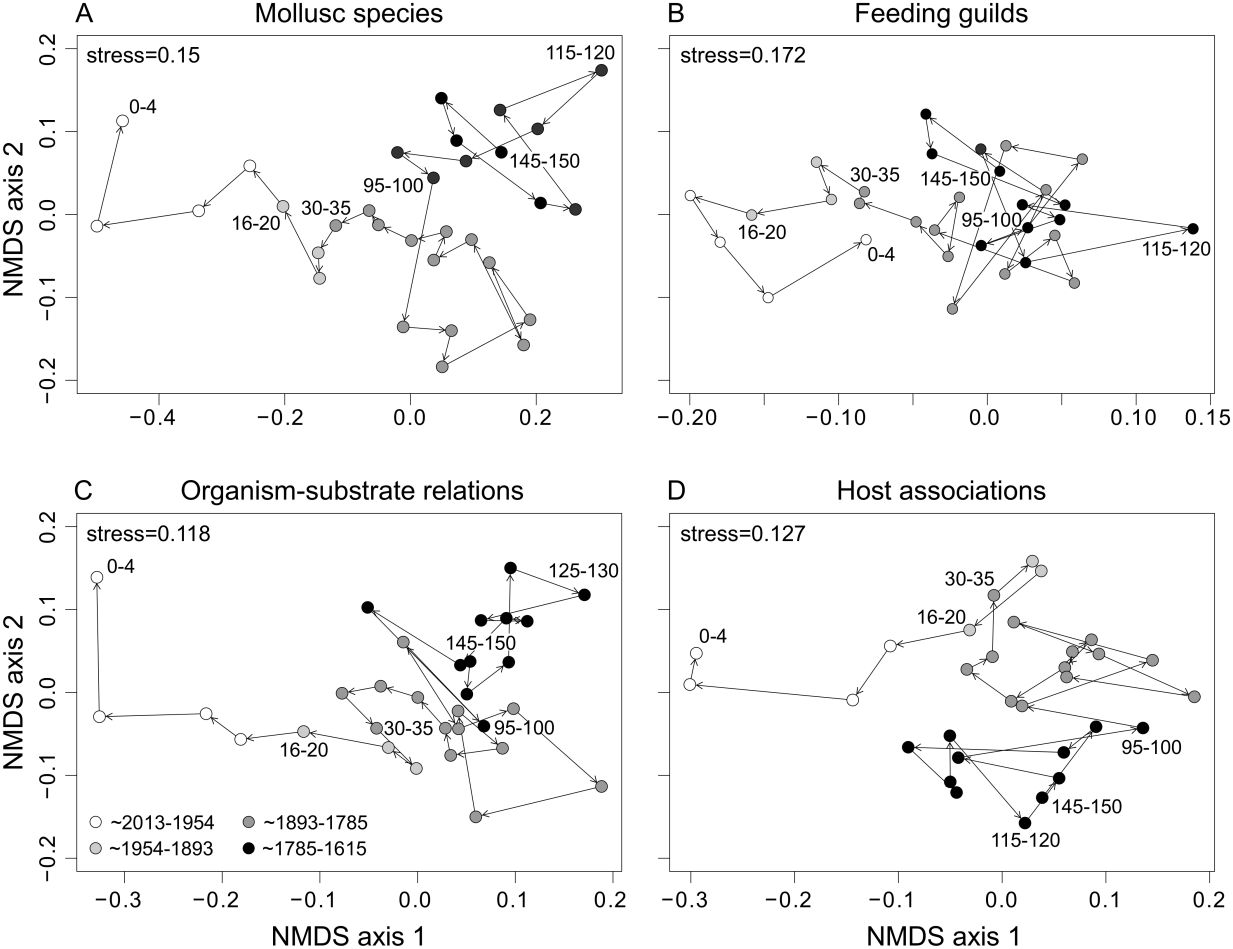
NMDS ordinations of the molluscan community. Ordinations are based on down-core species abundances (A), feeding guilds (B), organism-substrate relations (C), and host associations (D). Greyscale shadings of dots mark the four main molluscan community phases as listed in panel C.

NMDS ordinations reveal a marked community shift on the functional level as well as in species composition along the first axis at a core depth of about 40 cm, corresponding to the second half of the 19^th^ century (Fig 8A–8D). For feeding guilds, this shift is mainly influenced by the growing importance of scavengers and a simultaneous decline of most other feeding guilds. A conspicuous drop in epibionts and ectoparasites is the main driver for shifting organism-substrate relations between the subsurface and surface interval. Host associations display an overall decline in the uppermost 30 cm. This trend is reflected by the first axis in the ordination. The two groups separated along the second axis represent a shift towards a dominance of echinoderm associations in the intermediate section with a gradual decline of ascidian-associated species.

On the level of species composition (Fig 8A), the samples shallower than 40 cm show a strong and unidirectional change that can be divided into two phases: (a) from 40 to 20 cm (~1850–1950), with a peak of the host-associated bivalve *K*. *bidentata* and the infaunal gastropod *T*. *communis*; (b) from 20 cm to the surface (~late 20^th^ century), with a strong decrease of the latter, and an increase in the absolute abundance of several epifaunal (*N*. *pygmaeus*, *A*. *pespelecani*, *F*. *glaber*) and infaunal species (mainly *C*. *gibba* and *P*. *rhomboides*). The increments deeper than 40 cm (older than ~1900) are separated into two groups along axis 2: 40–95 cm (early 19^th^ century) and 95–150 cm (17-18^th^ century). The three dominant species (*K*. *bidentata*, *C*. *gibba*, *T*. *communis*) are almost equally abundant in the oldest group, but their relative abundance changes up-core (increasing in *K*. *bidentata* and *C*. *gibba*, decreasing in *T*. *communis*) driving the separation of these two deeper groups. The 115–120 cm sample is dissociated from the deep cluster due to a sharp peak in the relative abundance of *T*. *communis*.

#### Relations between environmental parameters and molluscan community change

For species composition and feeding guilds, strong negative correlations are evident between axis-1 scores of the NMDS (Fig 8) and a series of environmental variables, which form a cluster in the PCA (Fig 3): Ntot, PCB, and Pb (in addition, TOC and Zn for species composition). Changes in feeding guilds are furthermore significantly correlated with the concentrations of Cu und Ni. The concentrations of Ntot and PCB are those with the highest correlation coefficients and are also strongly correlated with changes in organism-substrate relations. (Table 3). No significant correlations were found between environmental variables and changes in host association. Likewise, for all aspects of community change, the correlation between NMDS-axis-2 scores and the measured environmental variables is insignificant. (Table 3).

**Table 3:** Correlations (Pearson’s r) between NMDS axis-1 and axis-2 scores and the investigated environmental variables.

	species		feeding guild		organism-substrate relation		host association	
	NMDS 1	NMDS 2	NMDS 1	NMDS 2	NMDS 1	NMDS 2	NMDS 1	NMDS 2
clay	0.30	0.73	0.43	-0.18	0.14	0.62	-0.03	-0.80
silt	-0.10	-0.79	-0.10	0.26	0.06	-0.60	0.20	0.81
sand	-0.61	0.02	**-0.83**	0.09	-0.57	-0.09	-0.46	0.18
gravel	-0.41	-0.03	-0.77	-0.27	-0.3	-0.15	-0.30	0.03
Hg	0.67	0.21	0.49	-0.31	0.61	0.48	0.29	-0.68
Cr	0.53	-0.13	0.53	0.14	0.53	-0.36	0.65	-0.19
Cu	0.79	-0.01	**0.84**	0.17	0.74	-0.02	0.67	-0.45
Ni	0.80	-0.11	**0.83**	-0.06	0.74	0.06	0.61	-0.62
As	0.26	0.46	0.29	0.44	0.15	0.25	0.30	-0.36
Cd	0.77	-0.57	0.54	-0.24	0.82	-0.45	0.71	-0.23
Pb	**-0.87**	-0.12	**-0.84**	0.14	-0.78	-0.21	-0.63	0.60
Zn	**-0.85**	-0.09	-0.78	0.19	-0.77	-0.27	-0.59	0.55
PAHs	-0.78	-0.33	-0.74	0.08	-0.66	-0.38	-0.51	0.72
PCB	**-0.89**	0.05	**-0.91**	-0.09	**-0.85**	-0.06	-0.78	0.37
TOC	**-0.81**	-0.06	-0.66	0.05	-0.76	-0.18	-0.60	0.58
N tot	**-0.96**	0.10	**-0.85**	-0.08	**-0.93**	-0.02	-0.82	0.48

Values are listed for each of the four ordinations shown in Fig 8.

Significant correlations in bold. P-values were corrected for multiple tests using the Holm-Bonferroni method.

**Fig 9:**
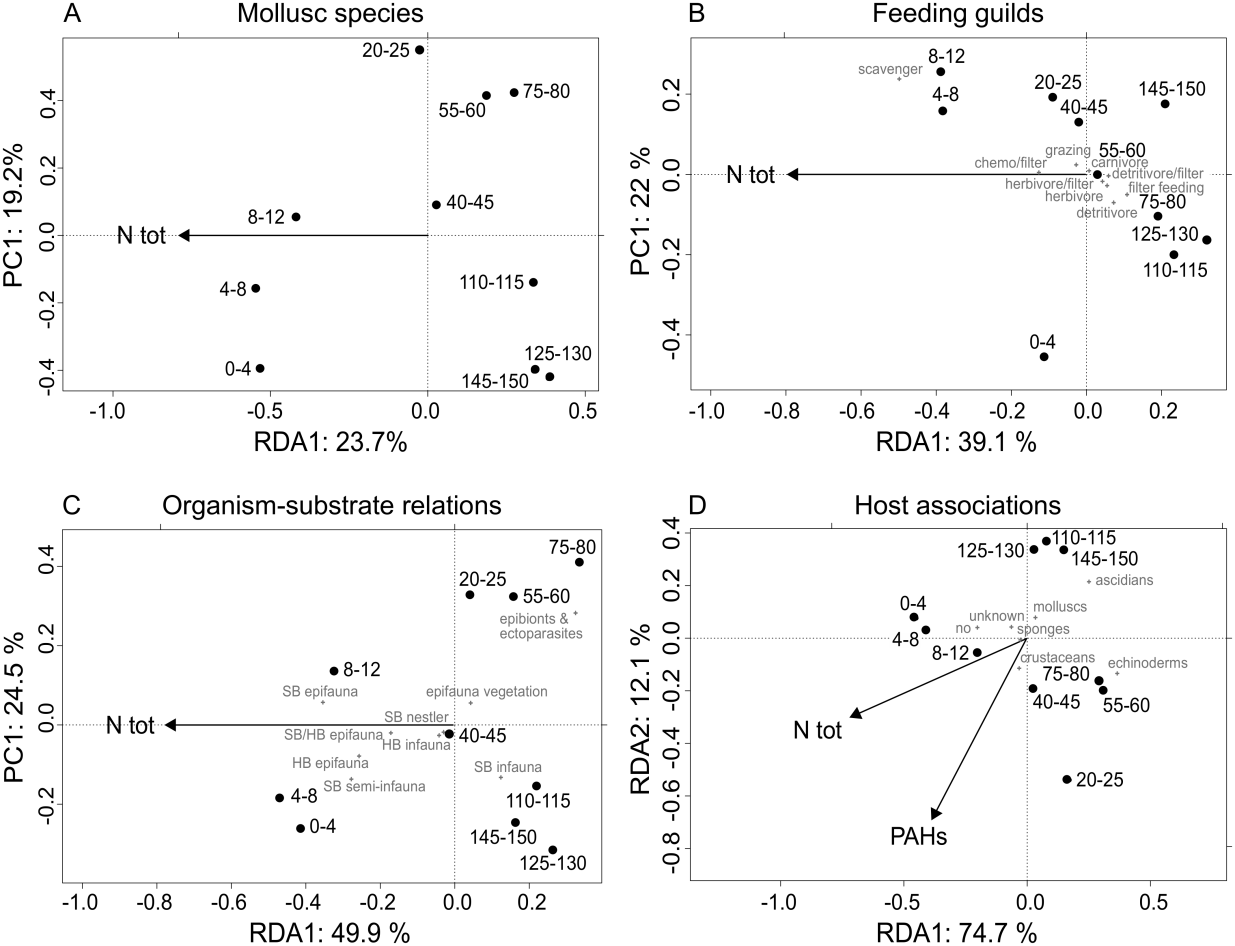
Redundancy analysis for down-core community changes in relation to environmental variables. Total nitrogen separates the topmost core intervals from the rest of the core and explains a significant amount of variation in relative abundance of species (A), feeding guilds (B), organism-substrate relations (C), and host associations (D). The second axis represent the first principal component in A-C because the stepwise selection selected one environmental variable only.

In the RDA, total nitrogen content explains the largest proportion of down-core variation in molluscan community composition. This variable effectively separates the assemblages of the uppermost core section (at least down to 12 cm depth) from the rest of the core. The same result is obtained for RDAs based on organism-substrate relations and feeding guilds. RDA for variations in host association provides PAH concentration as a second variable besides Ntot (Fig 9). However, other environmental variables with a close correlation to Ntot also explain a significant amount of variation if used for individual RDAs, including TOC, organic pollutants (PAH and PCB), and several metal concentrations (Mn, Fe, Ni, Cu, Cd, Zn) (Table 4).

**Table 4:** Results of individual RDAs.

Env. Var.	R^2^	F	Pr(>F)
N tot	0.485	7.525	**0.0001**
Mn	0.380	4.900	**0.002**
PCB	0.425	5.904	**0.003**
TOC	0.363	4.555	**0.004**
Pb	0.386	5.031	**0.005**
Cu	0.359	4.488	**0.006**
Zn	0.365	4.603	**0.006**
Ni	0.351	4.327	**0.008**
Cd	0.307	3.547	**0.009**
PAH	0.345	4.220	**0.009**
Fe	0.338	4.087	**0.010**
Li	0.296	3.367	**0.019**
P	0.275	3.041	**0.022**
Hg	0.233	2.424	0.059
sand	0.215	2.196	0.076
Al	0.213	2.169	0.079
Cr	0.184	1.809	0.116
gravel	0.147	1.383	0.209
clay	0.138	1.281	0.241
As	0.129	1.188	0.269
silt	0.101	0.895	0.429
C tot	0.078	0.674	0.765

Calculations are based on the forward/backward model selection. Listed are models with a single environmental variable (Env. Var.), R^2^ (explained variance), F-statistics and p-values from permutation tests.

## Discussion

### Sedimentology and associated community types

Despite the relatively long time-span covered by the two sediment cores from Panzano Bay, their sedimentological structure is very homogeneous, with silt and clay as the main sediment constituents almost uniformly distributed throughout the cores. Therefore, down-core changes in molluscan assemblages are driven by variables unrelated to sediment grain size. This observation makes the Panzano Bay cores a particularly interesting study object because the effects of pollution, eutrophication, hypoxia or fishing impact on community changes become more apparent. The variable and locally relatively high sedimentation rates in increments below 30 cm [23] probably reflect climate-driven, precipitation-dependent fluctuations of the Isonzo River water regime in pre-industrial times, with frequent floods and pronounced sedimentation pulses in the Gulf of Trieste [64, 65]. In addition, from the Middle Ages onwards, increased soil erosion due to land use and deforestation strongly augmented sediment delivery into the sea [66, 67]. These dynamics attenuated since the beginning of the 20^th^ century with the construction of several hydroelectric plants along the river and its tributaries, which lead to a reduction of sediment discharge into the sea [67, 68].

In the Bay of Panzano, reported sedimentation rates vary considerably depending on the distance and position relative to the Isonzo River mouth. In the GT2 core used for the assessment of Hg concentrations, sedimentation rates were estimated to be ~6 mm/y [39], in contrast to slower rate estimates in our cores (2–4 mm/y). This sampling location is ~2 km south-east from our position, and thus closer to the Isonzo River plume, has higher sand content, and does not contain any shell-rich interval in the uppermost part, pointing to a different sedimentary regime.

In the 1960s, the Gulf of Trieste was inhabited by communities typical of coastal terrigenous muds, dominated by *C*. *gibba*, *T*. *communis*, *A*. *pespelecani*, *Nucula nucleus* and *Dentalium dentalis* [38]. However, the clear distinction of a specific community type was complicated by the frequent dispersal of faunal elements from other population units, and by the fact that the whole area is subject to spatial and temporal variations in the type and rhythm of sedimentation [38, 69]. These sedimentary dynamics result not only from a strongly fluctuating riverine input, but also from storm-induced resuspension events (Bora and Sirocco winds) affecting the benthos and contributing to the formation of transitional communities [33, 70–72]. The aforementioned species are also well represented in our cores, but they undergo considerable proportional abundance changes from bottom to top. In contrast to the surveys from the sixties, benthic communities sampled since 1985 display a strong dominance of *C*. *gibba* [18]. This suggests that, in the Gulf of Trieste, mollusc communities are heterogeneous not only on a spatial, but also on a temporal scale.

Based on the results of the ordinations and the shell dating, we identified four core intervals, which are characterized by distinct molluscan shell communities.

### 16^th^-18^th^ century (150–95 cm)–pollution and effects of sedimentation rate

Lotze et al. [1] attribute this time span in the Adriatic Sea to the cultural period of market “development”, characterized by growing economies, the commercialization of resource use, onset of industrialization and, concerning fishing practices, mostly inshore fishing with selective and non-destructive gear (hook and line, light trawls towed by sail boats over soft bottoms). The molluscan community between 150 and 95 cm consists of many species that tolerate sedimentary instability and hypoxia, such as *C*. *gibba*, which has two distinct peaks in this interval, *A*. *pespelecani*, *Nuculana pella*, and the two naticids *Euspira macilenta* and *E*. *nitida*. On the other hand, species sensitive to disturbance, such as *K*. *bidentata* and *T*. *communis* [24], are also frequent, pointing to oscillations between periods of environmental instability and stable phases of recovery.

Absolute molluscan abundance drops to a minimum between 130 and 110 cm depth. Increased sedimentation rates during this period (~1 cm/year, Fig 6A) causing a higher dilution of shells by fine-grained sediment may be responsible for this trend and can be linked to the highly dynamic course of the Isonzo River in the 17^th^ and 18^th^ century. Driven by heavy floods and earlier human regulation of near-coast river sections, the Isonzo River left the Grado area and progressively migrated eastwards (Fig 1), slowly building up its modern deltaic system and inducing strong coastal erosion in areas no longer supplied by river sediments [56]. This century-long river migration process deeply reshaped the north-western Gulf of Trieste coastline and contributed to the variability and instability of sedimentary conditions in the Bay of Panzano.

The observed minimum in mollusc abundance between 130 and 110 cm core depth, however, could also be a consequence of elevated concentrations of several heavy metals (Ni, Cu, Cd, Cr), and exceptionally high concentrations of Hg. The latter can be traced back to the large mercury mine at the city of Idrija, located on the banks of a tributary of the Isonzo River. This epitome of early industrialisation was opened already in 1490 AD and washed the ore production wastes into Panzano Bay for almost five centuries [41, 73]. The extreme Hg concentrations (50–100 times more than NOAA or DM threshold concentrations) at about 125 cm core depth reflect the primitive and highly contaminative ore extraction methods in the early phases of mining [41, 74–76]. This inference is supported by the median shell age of *C*. *gibba* in this increment, which dates to the early 18^th^ century, thus corresponding to the early mining period. Covelli et al. [17], [39] linked the highest Hg concentrations found in two cores from Panzano Bay (~4 mg/kg in core AA1, 70 cm long, and ~23 mg/kg in core GT2, 320 cm long) to an intense Hg production period before World War I. These cores did not capture the earlier Hg contamination history, probably either due to the shorter core length (core AA1) or sparser sampling and higher condensation below 100 cm (core GT2). Importantly, the surficial Hg concentrations reported in Covelli et al. [17] for our sampling location (~ 8 mg/kg) are well in line with our own results. Hg is listed as one of the most toxic heavy metals to marine biota. It can strongly accumulate in tissues, trigger behavioural modifications and have negative effects on the settlement and survival of larvae and juveniles, already at concentrations much lower than those measured in our cores [77–80]. Similar effects are described for elevated concentrations of Cu and Cd, which also exceeded threshold values [61, 78, 81–85]. Ni and Cu, which by the PCA are grouped together with Hg and Cr, are identified as influential drivers of shifts in feeding guilds in the correlation analysis (Table 3). We note, however, that RDA did not detect the effects of Hg because its concentration sharply peaks just in a single increment (115–125 cm). The effects of toxic metals can be further masked by high mixing and time-averaging, which can reduce differences in abundance between increments with low and high concentrations of pollutants [23].

To summarize, communities in this time interval are characterized by strong oscillations in abundance of infaunal species differing in their sensitivity to disturbance, either due to temporarily increased input of toxic metals, variable sedimentation rates, or other causes such as decadal-scale climatic oscillations. The intense coastal fishing activities documented for this period, involving different kinds of gill and bottom trawl nets [51, 52, 86], might also have contributed to sediment instability and the scarcity of epifauna in our samples.

### 19^th^ century (95–30 cm)–shifts between colder and warmer conditions

In contrast to the earlier period, echinoderm-associated species (*K*. *bidentata* and *M*. *frilei*) increase and the gastropod *T*. *communis* declines in abundance (Fig 4). Additionally, some infaunal species (*Gouldia minima*, *Parvicardium exiguum*, and *N*. *pella*) decline, while epifauna (*N*. *pygmaeus*, *F*. *glaber*, *A*. *pespelecani*) becomes more numerous. The effective species richness of gastropods is significantly higher in the intermediate than in the deep section.

The lower part of the interval corresponding to the early 19^th^ century is marked by a conspicuous decrease in total molluscan abundance, reaching its minimum at ~1800 AD (~85 cm). Infaunal species such as *C*. *gibba*, *Abra* spp., *P*. *rhomboides* and the gastropod *T*. *communis* are most affected by this trend. Similarly as in the deepest core section, low abundances coincide with a high sedimentation rate (>1 cm/year), which in this context can be related to the Dalton temperature Minimum (“Little Ice Age”) entering one of its coldest phases in the early 19^th^ century. During this period, dropping temperatures and humid conditions led to substantially augmented river discharges, more extreme seawater temperature minima, high sedimentation and increased turbidity. Such conditions are well reflected in the foraminiferal record of our sediment cores and from other stations in the Adriatic, which in this period show abundance peaks of *Valvulineria complanata* (a species indicative for organically enriched mud belt environments) and a concurrent decrease in the frequency of epiphytic species [22, 87]. Thus, low molluscan abundances in the lower part of this core section may be due to a dilution effect generated by increased sediment supply. The concentrations of Ni, Cu, Cd and Cr peak here, and Hg still displays concentrations about 15 times higher than threshold values. As suggested by significant correlations between NMDS-axis scores and metal concentrations (Table 3), all these trace elements probably contribute to the overall abundance drop in molluscs via the various deleterious effects on behaviour, physiology, reproduction and larval development if present in high concentrations [77, 83, 88, 89].

The late 19^th^ century, captured by the upper part of this interval (below 30 cm), is characterized by an increase in the proportional abundance of *C*. *gibba*. This time interval coincides with rising sea-surface temperatures [23]. The shift from the *K*. *bidentata*-dominated state of the early 19^th^ century to the *C*. *gibba*-dominated state of the late 19^th^ century can thus be related to enhanced water-column stratification (driven by warming, which also induces the formation of mucilage) and subsequent occurrence of near-bottom hypoxia. *C*. *gibba* thrives under conditions of lowered oxygen and high nutrient concentrations and can dominate communities established after oxygen crises [90, 91]. Another species experiencing a notable increase during the late 19^th^ century (Fig 5) is the gastropod *N*. *pygmaeus*. This scavenger is the main driver for the detected community shift on the feeding-guild level. It tolerates low oxygen concentrations and benefits from the increasing food availability related to more eutrophic conditions and accompanying overall community growth [92]. Nonetheless, a real burst of the species occurs only in the overlying core section.

### Early 20^th^ century (30–16 cm)–high nutrient enrichment but rare hypoxia

The first half of the 20^th^ century is characterized by the emergence of a global economy and market, industrialisation processes in many economic areas, a strong population growth and a considerable increase of nutrient loads entering coastal seas in densely populated areas such as the northern Adriatic [1, 93]. The heavy metal burden in the sediments of Panzano Bay (Cu, Ni, Cr) decreases compared to the previous time interval, except for the constantly high Hg values and Pb and Zn, which experience a steep rise, in the case of Pb even slightly above TEL values. The rising concentration trends for Pb and Zn are negatively correlated with the content of the fine sediment fraction. This observation points to anthropogenic sources of enrichment, presumably related to the intensifying industrial activities in the port of Monfalcone, which, at the beginning of the century, saw also the establishment of a major shipyard [94]. The rising organic pollution (PCBs and PAHs) originated mainly from oil and coal combustion processes, with the Isonzo River and the Monfalcone port as the most plausible sources [22, 95, 96]. The low concentrations of PCBs in pre-industrial core intervals have to be interpreted as smearing-down effects induced by bioturbation, while in the case of PAHs, in addition to bioturbation effects, also natural sources (forest fires, natural oil seeps etc.) can be responsible for higher values in earlier times (see peak at ~85 cm, late 18^th^ century) [95]. Nutrient load in the northern Adriatic Sea progressively rises during the 20th century, as evidenced by the up-core nutrient and organic content increase (Fig 2). This rise is also reflected in the record of microfaunal and microfloral communities in our cores and in other sediment samples from this area; it was shown to be particularly marked between the 1930s and the 1970s [22, 93]. Increasing nutrient availability correlates with an abundance increase in several species. *K*. *bidentata* and *T*. *communis* reach their peak at 25–30 cm core depth. These species are sensitive to low oxygen concentrations, *K*. *bidentata* also due to the sensitivity of its echinoderm hosts [44, 97, 98]. Maxima in their proportional abundance and a concomitant decline of *C*. *gibba*, together with the direct documentation of low abundance of this species in samples collected in 1934 by Vatova [99], suggest that hypoxia events were rare during this period. For the Gulf of Trieste as well as for other parts of the northern Adriatic Sea, in fact only a few and small-scale mucilage or hypoxic events are reported for the first half of the 20^th^ century. Major mucilage events occurred either earlier (e.g. 1872) or, with increasing frequency, after the 1960s [44, 45, 100].

The small galeommatid bivalve *K*. *bidentata* lives as a filter feeder and detritivore near the substrate surface and establishes close associations with a series of host organisms [90, 101]. Brittle stars of the genus *Amphiura*, especially the species *A*. *filiformis*, are among its preferred hosts, supporting the bivalve not only in terms of food supply, but also with regard to reproduction and suitable habitat [90]. In the presence of this host species, which itself can reach densities of more than 1000 individuals per m^22^, densities of adult *K*. *bidentata* specimens may exceed those of the adults of all other bivalve species combined [101, 102]. Since eutrophication and the resulting higher food supply are suggested to be the main causes for the increase of this suspension-feeding ophiuroid [102], we conclude that the growing organic enrichment in the northern Adriatic towards the late 19^th^ / early 20^th^ century boosted *Amphiura* populations and thus, indirectly, also those of *K*. *bidentata*. This hypothesis is supported by the record of ophiuroidean arm ossicles and oral plates in the samples. The abundance of these skeletal remains displays a down-core trend remarkably similar to the one of *K*. *bidentata* (S1 Fig). Ophiuroidean arm ossicles, however, do no provide an unambiguous means for species identification, and oral plates of *Amphiura* and *Ophiothrix* are often hard to distinguish [103]. Nevertheless, the ossicle record may be taken as additional evidence for a strong relation between *K*. *bidentata* and *Amphiura* abundance trends. The increasing fishing effort during the early 20^th^ century may also have worked to the advantage of *Amphiura* populations by reducing predatory flatfish [104]. Another signal pointing to an increase of echinoderms are rising numbers of the eulimid gastropod *M*. *frilei*: this ectoparasite is strictly associated with echinoderms (holothurians) and its abundance trend almost parallels that of *K*. *bidentata* in our samples.

From their peak at 25–30 cm depth onwards, *K*. *bidentata* and *T*. *communis* drop in abundance. This decline, which leads to a near collapse of the species in the second half of the 20^th^ century, can be associated with increased fishing impact. Fisheries experienced considerable changes in the early 20^th^ century in the northern Adriatic. The number of boats involved in commercial coastal and offshore fishing grew, and many vessels were equipped with motors and mechanical winches. Traditional, selective types of nets were gradually replaced by more effective, but unselective and destructive gear, promoted by the growing power of motorized boats [1, 51]. The first impact of modern trawl fishing gear on the benthos could thus mark the reversal of a long-lasting upward trend in the abundance of many mollusc species characteristic of the 19^th^ century. The full industrialisation of fisheries and their severe impact on fish stocks and benthic fauna was only reached after the Second World War. Both world wars, on the other hand, marked a decisive cut in fishing activities, providing a recovery period for all affected species and habitats [51, 105]. This changing regime of disturbance and recovery is possibly also reflected in the growing importance of the epifaunal life habit, represented mainly by *N*. *pygmaeus* (Fig 7) above 25 cm (~1920). This scavenger apparently benefits particularly from the destructive effects of bottom-towed fishing gear on benthic organisms [106]. However, also a series of small and less abundant epifaunal mollusc taxa such as anomiids, *Ostrea* sp., *Hiatella arctica* and *Calyptraea chinensis* increase in abundance. These animals live firmly attached to hard substrates, and the availability of solid settling grounds in the form of dead and broken shells no doubt increases as a consequence of bottom trawling. The lower sedimentation rates of the last century, together with the war-related disruptions of fishing pressure, might additionally contribute to the success of these epibenthic species in colonizing the available benthic islands. Most of the found individuals, however, are juveniles, which points to a short duration of stable conditions not enabling maturation of the animals before re-burial.

### ~1950–2013 AD (16–0 cm)–high nutrient enrichment, frequent hypoxia, and the impact of bottom trawling

The uppermost sediment layer comprises the second half of the last century up until the time of sampling in 2013. Ordination patterns strongly separate assemblages in the upper four increments from the rest of the core, and RDA shows that the effects of increasing total nitrogen, correlating with other organic enrichments (TOC) and organic pollutants (PCBs, PAHs), are responsible for community changes. Eutrophication likely enhanced the recurrence of mucilage and hypoxic events, with more than ten such events documented for the Gulf of Trieste between the 1980s and the first decade of the 21^st^ century [44–46, 107]. Therefore, the high frequency of hypoxia and growing cultural eutrophication [93] and pollution represent the key factors of community change, although an enormous increase in fishing power probably also had major effects. In the northern Adriatic, new types of trawling gear such as the rapido trawl and the hydraulic dredge had a particularly severe impact on the benthos [51, 108]. Some local populations collapsed rapidly under this pressure, as was the case for natural banks of *Modiolus barbatus* in the Bay of Panzano,: they were heavily fished in the 1950s and disappeared altogether in the late 1960s due to overexploitation and habitat alteration [51, 109].

One of the victims of increased pressure from bottom trawling and hypoxia turns out to be *K*. *bidentata*, whose abundance drops rapidly. Trawling affects this bivalve both directly and indirectly by reducing the densities of its preferred host, the brittlestar *A*. *filiformis*. Echinoderms are also very vulnerable to low oxygen and high sulphide concentrations [92, 110, 111]. Mortalities, especially in brittle stars such as *A*. *filiformis*, may occur already at moderate hypoxic conditions because they leave the sediment when oxygen saturations drop below 10%, exposing themselves to predators [112]. The echinoderm-associated eulimid *M*. *frilei* follows the decline of *K*. *bidentata* and almost vanishes from the samples towards the surface. Echinoderm associations are not the only ones experiencing strong declines. The bivalve *M*. *subpictus*, associated with large epibenthic ascidians, reaches minima already during the early 20^th^ century and never recovers. Here, bottom trawling in particular affects the host and can damage the fragile bivalves directly.

Relative abundances of epi- and infaunal species increase in this core section to the expense of host-associated ones. However, their absolute numbers drop, in the case of *T*. *communis* or *A*. *nitida*, almost to zero. *T*. *communis* is described as hypoxia-sensitive [44, 113], and its decline probably reflects the increasing frequency of near-bottom hypoxic events in the second half of the 20^th^ century. *Abra* is more tolerant to oxygen depletion, but cannot survive severe hypoxia [114, 115]. Under hypoxic stress, the species may come to the surface, exposing itself to predators [116]. Generally, repeated hypoxia favours shallow burrowers over deep-burrowing species, a trend also visible in our cores [111].

The winners under these conditions are *C*. *gibba* and *N*. *pygmaeus*. Under frequently occurring near-bottom hypoxia, perpetual trawling and short recovery phases, the abundance trend of the opportunistic and hypoxia-tolerant *C*. *gibba* is almost diametrically opposed to that of *K*. *bidentata* and *T*. *communis*. The maxima in *C*. *gibba* abundances observed in present-day living assemblages point to outbreaks in the wake of seasonal hypoxic events [23, 117]. The high abundance of *C*. *gibba* also explains the increase in total mollusc abundance and the increase of infaunal and suspension-feeding life habits in the uppermost part of the core. Similar to *C*. *gibba*, the scavenging gastropod *N*. *pygmaeus* benefits from a rich food supply in the form of exposed, damaged or dead organisms available in heavily trawled areas or after oxygen deficiencies [106]. This species is mainly responsible for the growing importance of the epifaunal life habit and for scavenging as a significant feeding mode within the molluscan community. A dominance of infaunal species and a shift towards detritus-feeding and scavenging can be considered as typical features of highly disturbed areas. In addition to *C*. *gibba* and *N*. *pygmaeus*, other hypoxia-resistant species such as *A*. *pespelecani* [114, 118] and *N*. *nucleus* [115] increase in abundance or maintain their levels, underlining the importance of hypoxia as a major shaping force for the mollusc community in the recent past.

Species richness slightly (but not significantly) increases compared to the previous core section, in contrast to the loss of diversity usually reported for hypoxic conditions and high physical disturbance [111]. This increase can be interpreted as a mixing effect of alternating post-hypoxic, *C*.*-gibba*-dominated communities and recovered epifaunal-infaunal communities in a setting of repeated hypoxia and slow sedimentation. However, it may also be a true ecological signal. The species responsible for this trend are rare with only a single or few occurrences and comprise attached forms such as ostreids and anomiids as well as several free-living ones, typical either for detritic or soft bottoms (*Mimaclamys varia*, *F*. *glaber*, *Limaria* sp., *Laevicardium* sp.) or hard substrates (*Chiton* sp., *Crepidula* sp., *Diodora* sp., *Fusinus* sp.). All these species likely benefit from the increasing proportion of shell debris and broken serpulid tubes recorded in the surface sediments, and from low sedimentation rates. Comparable trends were described by Stachowitsch [118] and Nerlović et al. [117] for species such as *M*. *subpictus* or *Hiatella arctica*, whose populations rise after anoxic events due to an increased availability of dead shells as settling grounds. As in the previous core section, however, the generally small shell size of the specimens found points to early-stage epibenthic clumps, which were repeatedly disrupted in their further development by recurring trawling or hypoxia.

Overall, a rising number of species associated with detritic bottoms characterizes the surface core section. Nevertheless, abundances are clearly dominated by opportunistic and hypoxia-tolerant species such as *C*. *gibba*, *N*. *pygmaeus* or *A*. *pespelicani*. Species such as *T*. *communis*, *K*. *bidentata*, or *A*. *nitida*, typical representatives of the muddy bottoms in Panzano Bay that display high abundances in deeper core sections, strongly decline. Our interpretation is that the recent shift towards a detritic-bottom community, as favoured by more gradual and long-term environmental changes such as reduced sedimentation and rising eutrophication, is prevented by repeated short-term anthropogenic disturbances with direct and immediate impact (bottom trawling, hypoxia), benefitting species adapted to environmental instability.

## Conclusions

The molluscan community in the sediment cores from Panzano Bay experienced several shifts over the past ~500 years.

- Changing sedimentation rates throughout the cores, as reconstructed from median ages of dated *C*. *gibba* shells, influenced the molluscan shell record by reducing total abundances in those increments deposited during high-sedimentation periods. Down-core shifts in proportional abundance of functional groups and individual species, however, are attributable to a series of changing environmental and anthropogenic factors.
- In the deeper core sections, between the 16^th^ and 19^th^ century, a strong heavy metal contamination of the sediments, most notably by Hg, together with shifting climatic oscillations inducing changes in runoff and water-column stratification, drive changes in the molluscan communities, with dominance switching between *K*. *bidentata*, *T*. *communis*, and *C*. *gibba*.
- In the upper sections (from 1900 AD up to 2013), the combined effects of increasing eutrophication, more frequent hypoxic events and intensifying fishing pressure replace heavy metal contamination and climatic factors as the main drivers of community change and lead to the most pronounced shifts in molluscan community composition along the cores. In the early 20^th^ century, elevated nutrient input positively affects the communities, resulting in higher abundance and species richness. In the second half of the 20^th^ century, disturbances from fishing and hypoxia intensify, and opportunists such as *C*. *gibba* and *N*. *pygmaeus* benefit, whereas formerly dominant species such as *K*. *bidentata* or *T*. *communis* strongly decline. An infaunal life habit and scavenging emerge as effective strategies under these circumstances.
- In the lower part of the core, the molluscan assemblages are best described as the typical community of coastal terrigenous muds, with a high percentage of opportunistic species such as *C*. *gibba*, *N*. *pella*, *A*. *pespelecani*, and *Tellina distorta*. The presence of opportunists points to recurrent instability, either human-induced (Hg contamination, fishing) or natural (Isonzo River floods). The uppermost 30 cm (from 1900 AD onwards) bear signs for a transition towards a detritic-bottom community, with rising numbers of epifaunal and attached species. On one hand, this process seems to be anthropogenically triggered by providing more dead shells as settling grounds through bottom trawling and recurrent hypoxic events. On the other hand, trawling and hypoxia prevent the full development of a detritic communitiy and favour species adapted to unstable conditions, which occur again in high abundances.
- Results from the foraminiferal record of our sediment cores confirm eutrophication as the most important driver of community shifts [22]. The molluscan record, however, further reveals that heavy metal contamination of sediments, hypoxia and bottom trawling emerge as crucial factors shaping the macrobenthic communities together with the effects of a changing sedimentation regime and growing eutrophication. Down-core shifts in mollusc communities thus prove to be a sensitive and comprehensive proxy for past environmental changes, capturing factors such as hypoxia or fishing pressure that are difficult to detect in the foraminiferal record [111].
- Our historical ecology approach clearly explains the molluscan community shifts during the past ~500 years based on natural environmental trends and, above all, on strong anthropogenic impacts. These findings agree with and refine earlier findings [15] that identified anthropogenic impact as the crucial factor leading to the recent major shift in interglacial mollusc communities after their long-term endurance of climate-driven environmental changes. In addition, our analysis confirms the long history of human influence on marine ecosystems in the northern Adriatic Sea, extending back far beyond the Middle Ages. This perspective is no doubt valid for many other marine ecosystems and species in shallow coastal waters worldwide.

## Supporting information

S1 Dataset Abundance and functional categorisation of mollusc species. The dataset contains also max. size information for each species; last two rows list abundance of ophiuroid arm ossicles and oral plates.
(XLSX)

S2 Dataset Grain size distribution and geochemical data. (XLSX)

S1 Fig Down-core trends of ophiuroidean ossicles and *K*. *bidentata*. (TIF)
