# Supplementary figures and images for: Responses of molluscan communities to centuries of human impact in the northern Adriatic Sea
Source: PLoS One. 2017 Jul 19;12(7):e0180820. doi: 10.1371/journal.pone.0180820 (PMC5516990; doi:10.1371/journal.pone.0180820)

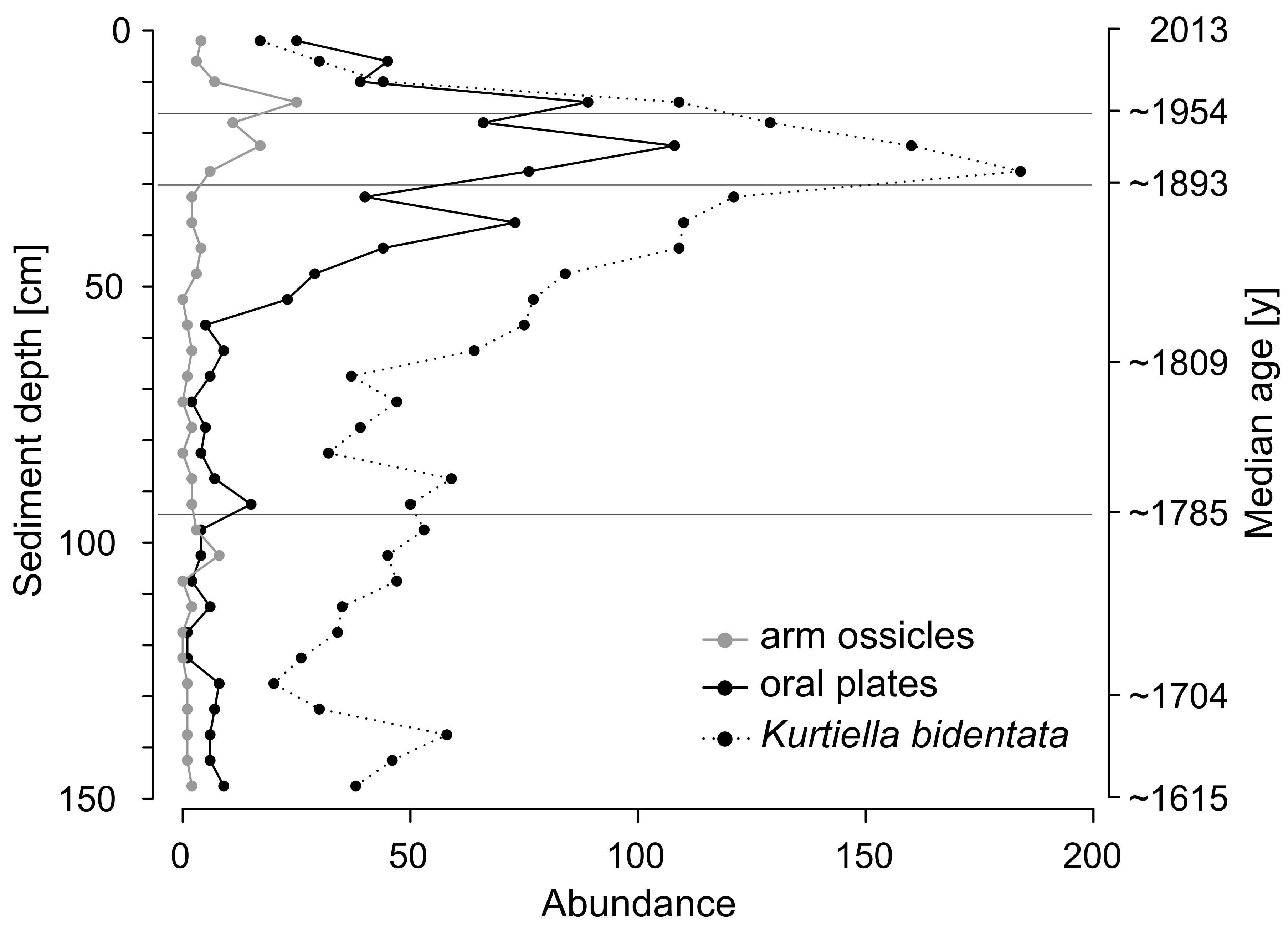

Supplement: S1 Fig — (TIF) [file pone.0180820.s003.tif]
